# Relationship Between Diet and Lifestyle and Liver Health: From the Most Recent Perspective

**DOI:** 10.3390/nu18010066

**Published:** 2025-12-25

**Authors:** Ioanna Panagiota Kalafati

**Affiliations:** 1 Department of Nutrition and Dietetics, School of Health Science and Education, Harokopio University of Athens, 17671 Athens, Greece; nkalafati@gmail.com; 2 Department of Nutrition and Dietetics, School of Physical Education, Sport Science and Dietetics, University of Thessaly, 42132 Trikala, Greece

Chronic liver diseases (CLDs)—particularly metabolic dysfunction-associated steatotic liver disease (MASLD)—are among the most prevalent and rapidly rising public health challenges worldwide. Despite affecting an estimated 25–38% of adults globally and encompassing a spectrum from simple steatosis to progressive liver injury, including inflammation, fibrosis, and cirrhosis, effective therapeutic modalities remain limited and predominantly lifestyle-based interventions are the cornerstone of management. This Special Issue of Nutrients, “Relationship Between Diet and Lifestyle and Liver Health: From the Latest Perspective,” brings together new insights that strengthen our understanding of how diet, physical activity, gut microbiota, body composition, and environmental exposures collectively shape liver health.

Tatsuta et al. highlight the importance of skeletal muscle mass relative to body weight as a meaningful marker of MASLD risk, underscoring the growing recognition that muscle–fat balance plays a central role in metabolic liver disease. Kim et al. report that polyphenol-rich freeze-dried plums can attenuate steatosis, oxidative stress, and gut barrier disruption in a MASLD model, reinforcing the therapeutic potential of functional foods. Complementing these mechanistic findings, Yang et al. demonstrate in a large cohort that higher physical activity levels significantly improve survival and reduce cirrhosis incidence, confirming physical activity as a key prognostic determinant.

Two accompanying reviews broaden our perspective. Jiménez-González et al. synthesize current evidence on gut microbiota dysregulation in MASLD, emphasizing the promise of AI-driven, multi-omics-based precision nutrition to guide individualized interventions. Nikolaou, Kalafati, and Dedoussis highlight the role of endocrine-disrupting chemicals and epigenetic mechanisms as emerging contributors to MASLD pathophysiology, illustrating the need to integrate environmental exposures into metabolic liver disease models.

Across these contributions, several themes clearly emerge: lifestyle behaviors are not peripheral but central drivers of MASLD; liver health is shaped by interconnected biological systems including muscle tissue, the gut–liver axis, and environmental toxicants; and the field is moving toward personalized, data-guided intervention strategies.

Future research must focus on (1) large-scale randomized trials of dietary and physical activity interventions, (2) mechanistic studies on the gut–liver-muscle axis, (3) the systematic incorporation of exposome science into MASLD risk assessment, and (4) the development and validation of precision nutrition tools supported by artificial intelligence.

This Special Issue contributes meaningful progress toward a more integrated understanding of MASLD and reinforces a central message: diet and lifestyle constitute powerful and actionable tools for preserving liver health and reducing the global burden of disease.

